# New Onset Anemia, Worsened Plasma Creatinine Concentration, and Hyperviscosity in a Patient With a Monoclonal IgM Paraprotein

**DOI:** 10.7759/cureus.41657

**Published:** 2023-07-10

**Authors:** Gregory D Sloop, Cheryl Moore, Gheorghe Pop, Joseph J Weidman, John A St. Cyr

**Affiliations:** 1 Pathology, Idaho College of Osteopathic Medicine, Meridian, USA; 2 Laboratory Services, Community Hospital of Monterey Peninsula, Monterey, USA; 3 Cardiology, Radboud University Medical Center, Nijmegen, NLD; 4 Internal Medicine, Independent Researcher, Columbia, USA; 5 Cardiac/Thoracic/Vascular Surgery, Jacqmar Inc., Minneapolis, USA

**Keywords:** blood flow, erythrocyte deformability, systemic vascular resistance response, paraprotein, igm, anemia, creatinine, viscosity, blood

## Abstract

A 76-year-old female followed closely for five years with IgM monoclonal gammopathy of uncertain significance developed anemia, worsened plasma creatinine concentration, and markedly elevated serum viscosity. This case illustrates the scope of pathology that can be caused by elevated blood viscosity. Our patient’s anemia was a homeostatic response to normalize systemic vascular resistance and resulted from activation of the systemic vascular resistance response. The elevated plasma creatinine resulted from decreased renal perfusion because of elevated blood viscosity. Recent insights in hemorheology (the study of blood flow) are discussed, namely the recent identification of preferential blood flow patterns and erythrocyte autoregulation of deformability. These insights confirm that blood viscosity is part of the “milieu intérieur.”

## Introduction

The effects of elevated blood viscosity are not always appreciated. Elevated blood viscosity is a significant abnormality because it reduces tissue perfusion, increases the risk of thrombosis, and increases vascular resistance [1]. The homeostatic mechanisms to maintain normal blood viscosity are even less well-known, having been described only recently [2]. Consequently, the anemia of chronic disease or inflammation is rarely recognized as the result of a homeostatic response. As a result, overtreatment of this anemia caused excess morbidity and mortality in the past [2]. Although it is accepted that conservative treatment of the anemia of chronic disease or inflammation is optimal because of the results from randomized prospective studies. It is not clear that the explanation for this is widely understood because the systemic vascular resistance response was only described recently and is not yet widely known.

## Case presentation

This 76-year-old female had been followed closely for five years with a diagnosis of IgM kappa monoclonal gammopathy of uncertain significance (MGUS). During that time, she reported episodes of weakness and dizziness which were worse with exertion. At times her serum had the consistency of corn syrup. Our patient’s most recent laboratory evaluation revealed new anemia with normal erythrocyte indices, worsened plasma creatinine, and elevated serum viscosity. Her hematocrit was 30.5%. Over the previous 29 months, it ranged from 36.2% to 42.4% (female normal range: 37.0-47.0 %). Her plasma creatinine was 2.0 mg/dL (normal: 0.52-1.04 mg/dL) and had ranged from 1.2 to 1.7 over the previous 29 months. There was never evidence of intrinsic renal diseases such as proteinuria, hematuria, pathological casts, or leukocytes on multiple urinalyses over the previous 29 months. The plasma albumin concentration was always within normal limits. Her serum viscosity was increased to 4.4 centistokes (cS) (normal: 1.5-1.9 cS). It had ranged from 2.1 cS to 3.8 cS over the previous 29 months.

In light of the worsened plasma concentration, her physicians planned to perform a renal biopsy to investigate the possibility of intrinsic renal disease until a laboratorian suggested the etiology could be increased blood viscosity and decreased renal perfusion. A bone marrow examination revealed normocellular marrow. Flow cytometry on the marrow revealed a monoclonal population of CD19+, CD20+ B lymphocytes, constituting 7.5% of nonerythroid nucleated cells, and a monoclonal population of kappa-restricted plasma cells, constituting 0.3% of nonerythroid nucleated cells. Both populations were negative *MYD88*. 

**Figure 1:**
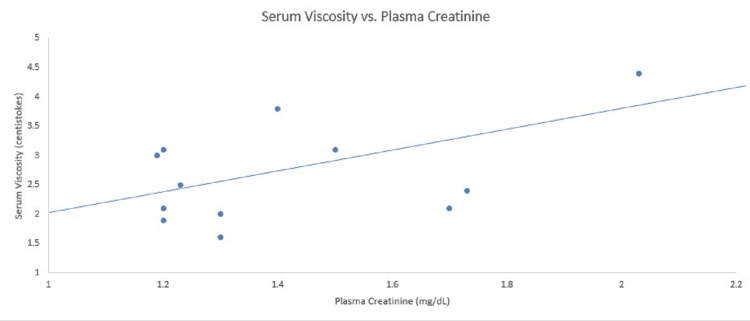
Relationship between serum viscosity and plasma creatinine concentration Serum viscosity is directly related to the plasma creatinine concentration.  The normal range for plasma creatinine is 0.52-1.04 mg/dL and serum viscosity is 1.5-1.9 centistokes.

**Figure 2:**
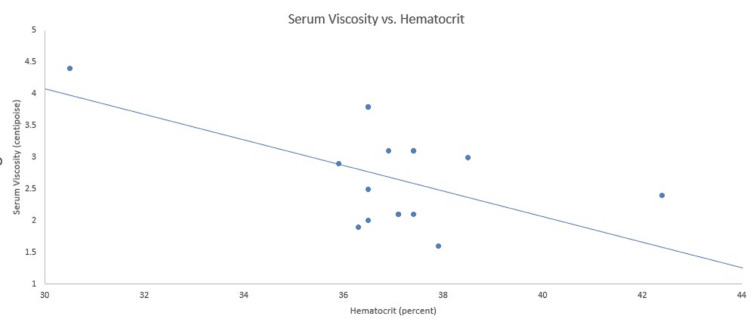
Relationship of hematocrit and serum viscosity The hematocrit is inversely related to serum viscosity. The normal range for serum viscosity is 1.5-1.9 centistokes and female hematocrit is 37.0-47.0%.

Serum viscosity, plasma creatinine, and hematocrit data from the previous 29 months are presented in Figures 1-2. As shown in Figure 1, the plasma concentration of creatinine is directly related to serum viscosity. Figure 2 shows that hematocrit is inversely related to serum viscosity.

## Discussion

IgM monoclonal gammopathy

A monoclonal IgM paraprotein can be present in MGUS, Waldenstrom’s hypergammaglobulinemia, and multiple myeloma. The influence of a molecule on viscosity is determined by its size, shape, and concentration. Because IgM is the largest immunoglobulin, with a molecular weight of 900-1,050 kDa, it can cause extreme elevations of whole blood, plasma, and serum viscosity. Our patient’s serum viscosity at times was higher than that of normal whole blood. Normal whole blood viscosity in females is 2.93 ± 0.43 centipoise or 2.76 ± 0.41 cS, assuming a specific gravity of blood of 1.0595 kg/m^3^.

IgM increases blood viscosity by two mechanisms: it directly increases serum and plasma viscosity, and it is large enough to act like glue and binds two erythrocytes simultaneously, thereby creating erythrocyte aggregates [3]. The electronegative surface charge of erythrocytes contributes to the zeta potential which separates them so that only the largest molecules cause aggregation. Erythrocyte aggregates increase blood viscosity by increasing the mass and inertia of the particles suspended in blood. They also increase vascular resistance because they must be disrupted before entering a capillary.

Blood viscosity is maintained as part of homeostasis

Blood viscosity is part of what the pioneering physiologist Claude Bernard called the “milieu intérieur” to describe the most fundamental parameters which are crucial to health such as temperature, pH, and osmolality. Blood viscosity must be normal for physiologic blood flow to occur. For example, normal wall shear stress causes endothelial production of nitric oxide and prostacyclin, molecules that have antiplatelet activity. Thus, blood viscosity must be within the physiologic range to maintain the antithrombotic phenotype of vascular endothelial cells.

The distinctive streamlines called “preferential flow patterns” develop only when blood viscosity and anatomy are normal [4]. These flows predictably develop every cardiac cycle and serve a physiologic purpose. For example, vortices develop in the aortic sinuses and cause the aortic valve to close. Blood flow in the heart follows preferential flow patterns. During diastole, blood from the left atrium is directed to the in-flow tract in the posterior aspect of the left ventricular cavity. Blood then turns around at the apex of the left ventricle with the aid of an intracavitary vortex, directing it toward the outflow tract.

Preferential flow patterns in the heart minimize the crossing of flow streamlines which minimizes friction and allow transport with the least energy cost. Loss of preferential flow patterns and crossing flow streamlines create pathologic vortices and friction which wastes energy. The energy dissipated from these vortices radiates as sound, resulting in a systolic flow murmur. Preferential flow patterns optimize blood flow.

**Figure 3:**
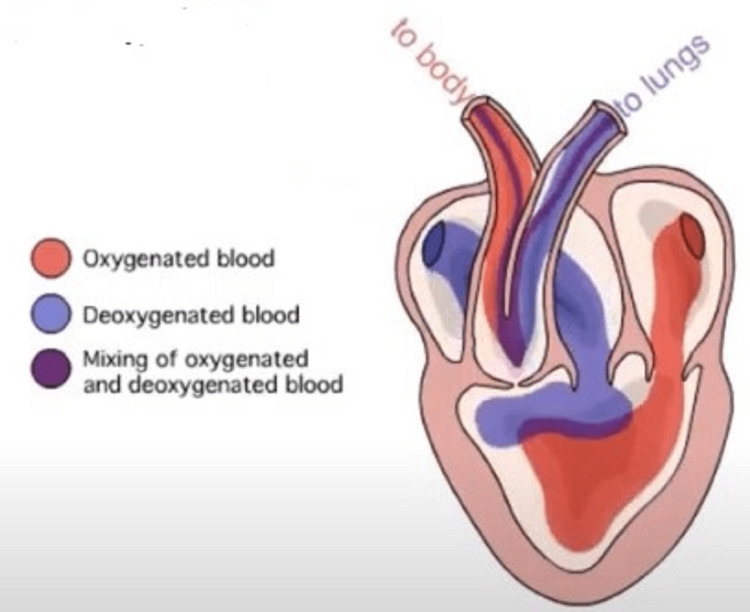
Preferential blood flow in the frog heart Preferential blood flow in the frog heart. Normal blood viscosity, cardiac anatomy, and function create a preferential flow pattern that allows the relative separation of oxygenated from deoxygenated blood.  Abnormally low blood viscosity will cause the streamlines to overshoot their targets so that the mixing of oxygenated and deoxygenated blood increases.  High blood viscosity will cause incomplete filling of the ventricle. As a result, the filling of the ventricular apex, that part of the heart which is physically the most distant from the layer of deoxygenated blood, is decreased. The physical separation of oxygenated and deoxygenated blood is diminished, increasing the mixing of the two. Used with permission from iWorx Systems Inc.

Perhaps the most impressive example of a preferential flow pattern is seen in the three-chambered frog heart. Preferential flow patterns allow the relative separation of oxygenated and deoxygenated blood in the frog heart (Figure 3). Abnormal blood viscosity will increase the mixing of oxygenated and deoxygenated blood in the frog heart and impair physical performance.

The requirement for normal blood viscosity for the development of preferential flow patterns is seen in coronavirus disease 2019 (COVID-19). Inflammation is a hyperviscous state. A recent study showed that the preferential flow pattern in the left ventricular outflow tract was lost in a population of COVID-19 patients with normal coronary arteries, normal wall motion on echocardiography, and no laboratory evidence of myocardial damage but with a thrombus in the left ventricle. Progression through the normal phases of blood flow was delayed, flow velocity was slower, the direction of flow was abnormal, and pathologic turbulence was observed. The abnormalities persisted even after the thrombus was lysed. The authors concluded that normal ventricular wall motion was insufficient to maintain the preferential flow pattern in the left ventricular outflow tract and its loss might lead to intracavitary thrombosis. They speculated that the loss of the preferential flow pattern was caused by increased blood viscosity [5].

Control of blood viscosity

The importance of blood viscosity in homeostasis is demonstrated by the multiple mechanisms that sense and control it. Three aspects of blood viscosity are sensed: systemic vascular resistance, shear stress, and shear rate. Viscosity is controlled by varying the hematocrit and erythrocyte deformability. This control is so robust that abnormalities of blood viscosity are rare and often not recognized as such. However, complications of hyperviscosity can develop during the brief period before compensation develops or if compensation is incomplete.

Hematocrit is a strong determinant of blood viscosity. The anemia in this case was compensatory to normalize whole blood viscosity. It resulted from the activity of the systemic vascular resistance response (SVRR) [2]. Anemia lowers blood viscosity thereby decreasing the risk of thrombosis and other complications of hyperviscosity.

The SVRR was described in 2015. It is activated by stretching mechanoreceptors in the left ventricle which occurs with increased systemic vascular resistance or increased intravascular volume. Stretching of these mechanoreceptors releases BNP from cardiomyocytes which causes vasodilation and natriuresis. Also, red blood cell mass is reduced by several mechanisms. Thus, the SVRR causes anemias of chronic disease and inflammation, renal failure, and heart failure.

An unusual cause of increased intravascular volume and activation of the SVRR is the anemia of spaceflight. Upon release from gravity, fluid from gravity-dependent areas shifts into the intravascular volume, activating the SVRR. Upon return to Earth, intravascular volume is restored more quickly than red cell mass, resulting in the transient anemia of spaceflight [6].

The SVRR is the physiologic antagonist of the renin-angiotensin-aldosterone system (RAAS). Input into the RAAS comes from the carotid sinus, which senses wall shear stress [7]. The dilatation of the carotid sinus causes a preferential flow pattern that amplifies wall shear stress, making it sensitive to changes in small changes in wall shear stress. Neural output from the carotid sinus projects to the brainstem via the glossopharyngeal nerve (CN IX) where it modulates neural input into the kidney.

The RAAS causes vasoconstriction and sodium retention and increases red blood cell mass. The most dramatic example of the effect of RAAS on erythropoiesis is posttransplant erythrocytosis. This is defined as a hematocrit >51% or a hemoglobin concentration >17 mg/dL after renal transplantation. Approximately 60% of patients experience malaise, headache, lethargy, and dizziness. Ten to 30% of patients develop thrombotic events. Blockade of the RAAS is the most effective therapy.

IgM molecules activate the SVRR by both mechanisms: they increase blood viscosity and systemic vascular resistance and, because they are osmotically active, cause a shift of extravascular fluid to the intravascular space.

**Figure 4:**
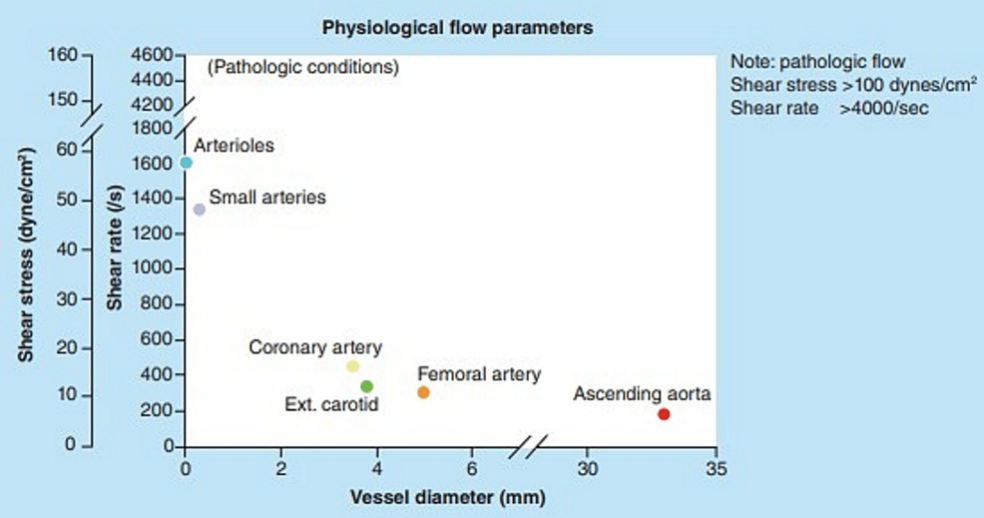
Shear rate and wall shear stress in various arteries and arterial beds Shear rate and wall shear stress in various arteries and arterial beds. Reproduced from reference [8]. Reuse permitted under CC BY-SA 4.0.

The third aspect of the control of blood viscosity is the sensing and response to shear rate by erythrocytes. The shear rate of blood can be thought of as the velocity gradient within it. In a column of flowing blood, the velocity is highest in the center and slowest against the vessel wall. The shear rate is highest in small arteries and arterioles which are small vessels that contain high-velocity flow (Figure 4) [8].

A high shear rate causes erythrocytes to deform physically. It is now established that erythrocytes increase their deformability in response to high shear [9]. Pliable erythrocytes decrease the resistance to flow which decreases blood viscosity. Increased deformability also facilitates erythrocyte passage through capillaries. The erythrocytic response to shear rate may play a role in the Fåhræus-Lindqvist effect, i.e., the progressive decrease in blood viscosity as tube diameter decreases from slightly greater than 0.3 mm to 0.04 mm.

In the left ventricular outflow tract and aortic arch, where blood velocity is high and the direction of flow streamlines change, increased blood viscosity is needed to minimize energy loss to turbulence. The risk of developing turbulence in curved tubes is described by the Dean number. Turbulence develops at Dean numbers which are much lower than Reynolds numbers, which describe the chance of developing turbulence in straight tubes. Both the Dean and Reynolds numbers are inversely related to blood viscosity.

It is possible that erythrocyte deformability decreases and blood viscosity increases in the left ventricular outflow tract and aortic arch because of the activity of prostaglandin E2 (PGE2). This molecule has been shown to decrease erythrocyte deformability in vitro [10]. PGE2 is synthesized by alveolar epithelial cells, and its concentration in bronchoalveolar lavage fluid is 100X higher than the concentration in pulmonary arterial and peripheral venous blood [11]. Its activity in the bloodstream is limited by its short half-life, 10 to 30 s.

**Figure 5:**
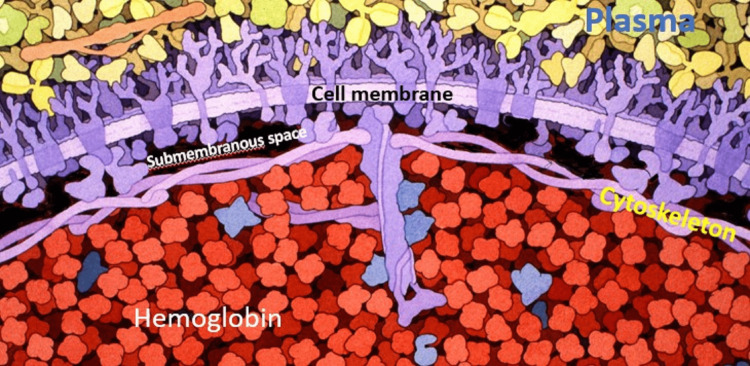
Artist rendering of plasma, erythrocyte cell membrane, submembranous space, cytoskeleton, and bulk cell compartment Artist rendering of plasma, erythrocyte cell membrane, submembranous space, cytoskeleton, and bulk cell compartment.  The submembranous space is estimated to be 5 nm thick, which allows unimpeded diffusion of inorganic ions, which range from tens to hundreds of pm in diameter, and adenosine triphosphate (ATP), which measures 1.4 nm in diameter, throughout the space. The average thickness of the cytoskeleton in the central portion of the erythrocyte is 110 nm (range 88-128 nm) and 54 nm (range 36-72 nm) at the periphery. This is thick enough to sequester hemoglobin molecules, which measure 5 nm in diameter, in the bulk cell compartment. Structures are not depicted to scale. Modified from an original work by David S. Goodsell entitled "Biosites: Red Blood Cell" (2005). Reuse permitted under CC BY-SA 4.0.

PGE2 may bind to erythrocytes during passage through the pulmonary microcirculation. This will activate a non-selective cation channel [12], increasing the calcium ion concentration in the submembranous space between the erythrocyte cell membrane and cytoskeleton (Figure 5).

The submembranous space is hemoglobin-free which optimizes the diffusion of adenosine triphosphate (ATP) and inorganic ions. It also permits rapid changes in ion concentrations and quicker responses to these changes, which would be much slower if responses required a change in the ion concentration of the entire corpuscular volume. Whole-corpuscle changes in ion concentrations take minutes to more than one hour to proceed to completion in vitro. Such a response is inadequate when one considers that an erythrocyte takes roughly 60 seconds to complete a circuit through human circulation.

Once in the submembranous space, calcium ions activate the Gardos channel, a calcium-activated potassium channel, causing the efflux of potassium ions and water. The water channel aquaporin-1 makes the erythrocyte cell membrane permeable to water. There are estimated to be 200,000 of these channels in the cell membrane. Loss of intracellular water increases erythrocyte cytoplasmic viscosity and temporarily decreases erythrocyte deformability. This increases blood viscosity. An even greater increase in blood viscosity is necessary when a sympathetic response increases cardiac output and peak blood velocity. This is accomplished by epinephrine, which also decreases erythrocyte deformability in vitro [10].

The loss of erythrocyte deformability is reversed by the many calcium pumps in the erythrocyte cell membrane. Erythrocytes are estimated to have between 1,000 and 4,500 calcium pumps, in contrast to 400 sodium channels. Erythrocytes encounter progressively higher shear as vessel diameters in the arterial system decrease. This shear activates Piezo1, a nonspecific cation channel. The movement of cations into the erythrocyte is followed by water so that by the time an erythrocyte reaches the capillaries, the mean corpuscular volume (MCV) of erythrocytes is 3.1 fL greater than that of erythrocytes in the venous circulation [13]. There are approximately 10^11^ molecules of water in 3.1 fL. Since approximately 10^9^ water molecules can pass through one aquaporin channel per second, that number of water molecules can pass through 2 x 10^6^ channels in less than one second.

Presumably, water molecules are squeezed from erythrocytes as they pass through capillaries, which have a diameter smaller than erythrocytes. Thus, in the course of their journey through the circulation, erythrocyte deformability changes in response to the local milieu, resulting in constantly changing blood viscosity in vivo.

Like atherosclerotic coronary artery disease (ASCVD), Sir William Osler described aortic valve stenosis as having a low prevalence in the early twentieth century. The prevalence of both has increased. Both conditions share risk factors, and viscosity plays an etiologic role in both [14]. Inhibitors of the enzyme cyclooxygenase decrease synthesis of PGE2 and may eliminate the loss of deformability following passage through the pulmonary circulation in vivo. This will decrease blood viscosity and increase peak blood velocity and erythrocyte momentum. These changes will increase the energy transferred to the aortic valve when blood interacts with it in systole, accelerating its degeneration. Consistent with this, the use of celecoxib, a cyclooxygenase-2 (COX-2) inhibitor, is associated with an increased risk of aortic valve stenosis [15]. The COX-2 pathway is used in the constitutive production of PGE2 in the human lung [16].

It is possible that blood viscosity in vivo is tailored to the local flow environment by modulating erythrocyte deformability: increased deformability reduces blood viscosity in the microvasculature and decreased deformability increases blood viscosity in the left side of the heart and proximal aorta. At this time, autoregulation of blood viscosity in the left ventricular outflow tract and low-shear environments of elastic and conducting arteries has not been demonstrated. This will require echocardiography or 4D flow MRI imaging of the aortic arch and thoracic aorta, before and after the inhibition of cyclooxygenase. A COX-2 inhibitor should increase blood velocity and turbulent kinetic energy in the aortic arch. 

Complications of blood hyperviscosity

Myocardial infarction, hypertension, diabetes mellitus type 2, metabolic syndrome, and aortic stenosis are examples of diseases of superabundance, i.e., diseases whose prevalence has increased because of the higher standard of living afforded by industrialization. Increased blood viscosity probably plays a pathogenic role in all [17]. A higher standard of living increases blood viscosity because better nutrition allows higher plasma protein concentrations and the elimination of parasites that cause anemia such as hookworm and whipworm.

Increased blood viscosity causes pathology via three mechanisms: decreased tissue perfusion, increased risk of thrombosis, and increased systemic vascular resistance. Experimentally, an increase in blood viscosity causes a threefold decrease in blood flow [1]. Increased blood viscosity and concomitantly decreased perfusion of the central nervous system and musculature are straightforward explanations for our patient’s dizziness and weakness.

Increased blood viscosity is also a likely cause of our patient’s chronically elevated serum creatinine concentration. Creatinine is a breakdown product of creatine phosphate, a source of high-energy phosphate in times of greater demand. It is filtered and secreted by the kidney but not reabsorbed. Thus, plasma creatinine concentrations are directly related to renal blood flow. The creatinine concentration is criticized as a marker of renal function because it is elevated only after a substantial number of glomeruli are lost. However, serial testing in a patient without intrinsic renal disease provides a reasonable marker of renal perfusion.

Diminished renal blood flow directly increases blood viscosity by increasing the blood concentrations of organic ions such as uric acid, homocysteine, trimethylamine N-oxide, and phenylacetylglutamine. These molecules are actively secreted from the blood across the renal tubular epithelium into the urine. Decreased renal perfusion allows their accumulation in the blood. Organic ions are larger and have a lower charge density than the small inorganic ions, sodium, and chloride, which normally determine the ionic strength of blood. Thus, organic ions decrease the ionic strength of plasma, thereby reducing the zeta potential. When it decreases, smaller molecules such as IgG can cause erythrocyte aggregation [18]. Those molecules whose concentration is increased in renal failure are known as “protein-bound uremic toxins.” Those which are products of the microbiome are called “gut-derived uremic toxins" [1]. 

Sluggish blood flow, which pathologist Rudolph Virchow identified as a cause of thrombosis, is simply a manifestation of increased blood viscosity [1]. The role of acute hyperviscosity in thrombosis became clear in severe COVID-19 [19]. An increased incidence of thrombosis in relatively young patients in intensive units on prophylactic anticoagulation was noted early in the pandemic. In inflammation, increased blood viscosity is principally caused by hyperfibrinogenemia and hypergammaglobulinemia.

Insufficient blood viscosity is one cause of high-output heart failure. As discussed, blood viscosity contributes to systemic vascular resistance. Lack of resistance results in abnormally high cardiac output, turbulence, and hemolysis despite systemic vasoconstriction.

Bleeding is often described as a component of hyperviscosity syndrome. However, bleeding is caused by the nonspecific binding of paraproteins to platelets and is not a manifestation of hyperviscosity itself.

Blood viscosity and accelerated atherothrombosis

Blood viscosity in the upper part of the statistically normal range was shown prospectively to be a strong risk factor for myocardial infarction [20]. This elevation is caused by erythrocyte aggregation fostered by LDL particles and fibrinogen. High-density lipoprotein (HDL) particles antagonize erythrocyte aggregation caused by LDL and decrease blood viscosity [1]. Erythrocyte aggregates and increased blood viscosity create larger areas of slower blood flow in areas of low shear in arteries, which are the regions susceptible to atherogenesis. This fosters mural thrombosis. According to the thrombogenic theory of atherogenesis, the organization of a mural thrombus results in an atherosclerotic plaque. Thus, one process, thrombosis, causes both the thrombotic complications of atherosclerosis such as myocardial infarction and stroke, and the underlying plaque. The thrombogenic theory of atherogenesis has recently been reviewed [3].

## Conclusions

Blood hyperviscosity should be considered in the differential diagnosis of normocytic, normochromic anemia and elevations of the creatinine concentration. In patients with anemia of chronic disease or inflammation, one should be aware that the anemia is caused by a compensatory mechanism to prevent the detrimental effects of potentially hyperviscous blood. Thus, this anemia should be treated conservatively.
